# Statin Use Is Associated with Reduced Risk of Haematological Malignancies: Evidence from a Meta-Analysis

**DOI:** 10.1371/journal.pone.0087019

**Published:** 2014-01-31

**Authors:** Xiao Yi, Wei Jia, Yin Jin, Shang Zhen

**Affiliations:** Department of Hematology, Tongji Hospital, Tongji Medical College, Huazhong University of Science and Technology, Wuhan, China; Sanjay Gandhi Medical Institute, India

## Abstract

**Background:**

Several observational studies have shown that statin use may modify the risk of haematological malignancies. To quantify the association between statin use and risk for haematological malignancies, we performed a detailed meta-analysis of published studies regarding this subject.

**Methods:**

We conducted a systematic search of multiple databases including PubMed, Embase, and Cochrane Library Central database up to July 2013. Fixed-effect and random-effect models were used to estimate summary relative risks (RR) and the corresponding 95% confidence intervals (CIs). Potential sources of heterogeneity were detected by meta-regression. Subgroup analyses and sensitivity analysis were also performed.

**Results:**

A total of 20 eligible studies (ten case-control studies, four cohort studies, and six RCTs) reporting 1,139,584 subjects and 15,297 haematological malignancies cases were included. Meta-analysis showed that statin use was associated with a statistically significant 19% reduction in haematological malignancies incidence (RR = 0.81, 95% CI [0.70, 0.92]). During subgroup analyses, statin use was associated with a significantly reduced risk of haematological malignancies among observational studies (RR = 0.79, 95% CI [0.67, 0.93]), but not among RCTs (RR = 0.92, 95% CI [0.77, 1.09]).

**Conclusions:**

Based on this comprehensive meta-analysis, statin use may have chemopreventive effects against haematological malignancies. More studies, especially definitive, randomized chemoprevention trials are needed to confirm this association.

## Introduction

Hematologic malignancies, including three major groups: leukemia, lymphoma, and plasma cell neoplasms, derive from cells of the bone marrow and the lymphatic system [1]. In general, the overall incidence of hematological malignancies appears to be rising in Western countries, however, it is very difficult to describe their epidemiological behavior in a consistent and uniform way. In the USA, the number of estimated new cases of hematological malignancies in 2011 was 140,310 and it was predicted to have 53,010 deaths due to hematological malignancies [2].

3-hydroxy-3-methylglutaryl-coenzyme A reductase inhibitors (statins) are used for primary and secondary prevention of cardiovascular diseases, and their efficacy on cardiovascular events has been proven irrefutably for both reduction of morbidity and mortality [3], [4]. Statins are also found to be associated with decreased risk of certain cancers [5], [6] and reduce cancer-related mortality [7]. In vitro and animal studies have shown that statins have anti-proliferative, pro-apoptotic, anti-angiogenic and immunomodulatory effects, which prevent cancer development, growth, and metastasis [8]–[12].

Several randomized controlled trials(RCTs) and epidemiologic studies have evaluated the association between statin use and the risk of haematological malignancies; however, the existing results are inconsistent. To better understand this issue, we carried out a meta-analysis of existing RCTs and observational studies that investigated the association between statin use and the risk of developing haematological malignancies.

## Methods

### Literature Search

This meta-analysis was conducted following the guidance provided by the Cochrane Handbook and was reported according to the Meta-analysis of Observational Studies in Epidemiology (MOOSE)guidelines [13]. A systematic literature search of PubMed, Embase, and Cochrane Library Central database was conducted for all relevant articles investigating the effect of statin use on the risk of haematological malignancies between January 1966 and July 2013. Search terms included: “hydroxymethylglutaryl-CoA reductase inhibitor(s)” or “statin(s)” or “lipid-lowering agent(s)” and “tumour(s)” or “cancer(s)” or “neoplasm(s)” or “malignancy(ies)” and “lymphatic” or “haematopoietic” or “hematopoietic” or “leukemia” or “lymphoma” or “haematological” or “blood” or “multiple myeloma”. In addition, we reviewed the reference lists from all relevant articles to identify additional studies.

### Study Selection

We first excluded all irrelevant papers based on the titles and abstracts of the articles, and then the full texts of the remaining articles were read to determine whether they contained information on the topic of interest. Studies considered in this meta-analysis were either RCTs or observational studies that met the following inclusion criteria: (i) evaluated and clearly defined exposure to statins, (ii) reported haematological malignancies incidence and (iii) presented odds ratio (OR), relative risk (RR), or hazard ratio (HR) estimates with its 95% confidence interval (CI), or provided data for their calculation. There were no restrictions of origin, study size, language or publication type. Exclusion criteria was (i) lack of available data (ii) reviews, editorials, comments, reports from scientific sessions or discussions.When there were multiple publications from the same population, only data from the most recent comprehensive report was included.

### Data Extraction

Data was independently abstracted onto a standardized form by two authors. The following data was collected from each study: name of the first author, publishing time, study design, country of the population studied, study period, follow-up time, statin type, RR, OR, HR and their 95% CIs, confounding factors for matching or adjustments.

### Statistical Analysis

In our meta-analysis, we pooled data using the fixed or random effect models depending on heterogeneity between studies. Heterogeneity was assessed using the Cochran Q and I^2^ statistics. For the Q statistic, a P value<0.10 was considered statistically significant for heterogeneity; for the I^2^ statistic, heterogeneity was interpreted as absent (I^2^∶0%–25%), low (I^2^∶25.1%–50%), moderate (I^2^∶50.1%–75%), or high (I^2^∶75.1%–100%) [14]. When substantial heterogeneity was detected, the summary estimate based on the random-effect model (DerSimonian–Laird method) [15] was reported, which assumed that the studies included in the meta-analysis had varying effect sizes. Otherwise, the summary estimate based on the fixed-effect model (the inverse variance method) [16] was reported, which assumed that the studies included in the meta-analysis had the same effect size.

The overall analysis including all eligible studies was performed first, and subgroup analyses were performed according to (i) study design(observational studies, RCTs), (ii) study location(Western countries, Asian countries), (iii) study setting (population-based, hospital-based), (iv) subtypes of haematological malignancies(leukemia, lymphoma, multiple myeloma) to examine the impact of these factors on the association. To test the robustness of association and characterize possible sources of statistical heterogeneity, sensitivity analysis was carried out by excluding studies one-by-one and analyzing the homogeneity and effect size for all of rest studies. To better investigate the possible sources of between-study heterogeneity, a meta-regression analysis was performed [17]. Publication bias was assessed using Begg and Mazumdar adjusted rank correlation test and the Egger regression asymmetry test [18], [19]. All analyses were performed using Stata version 11.0 (StataCorp, College Station, TX).

## Results

### Search Results

**Figure 1:**
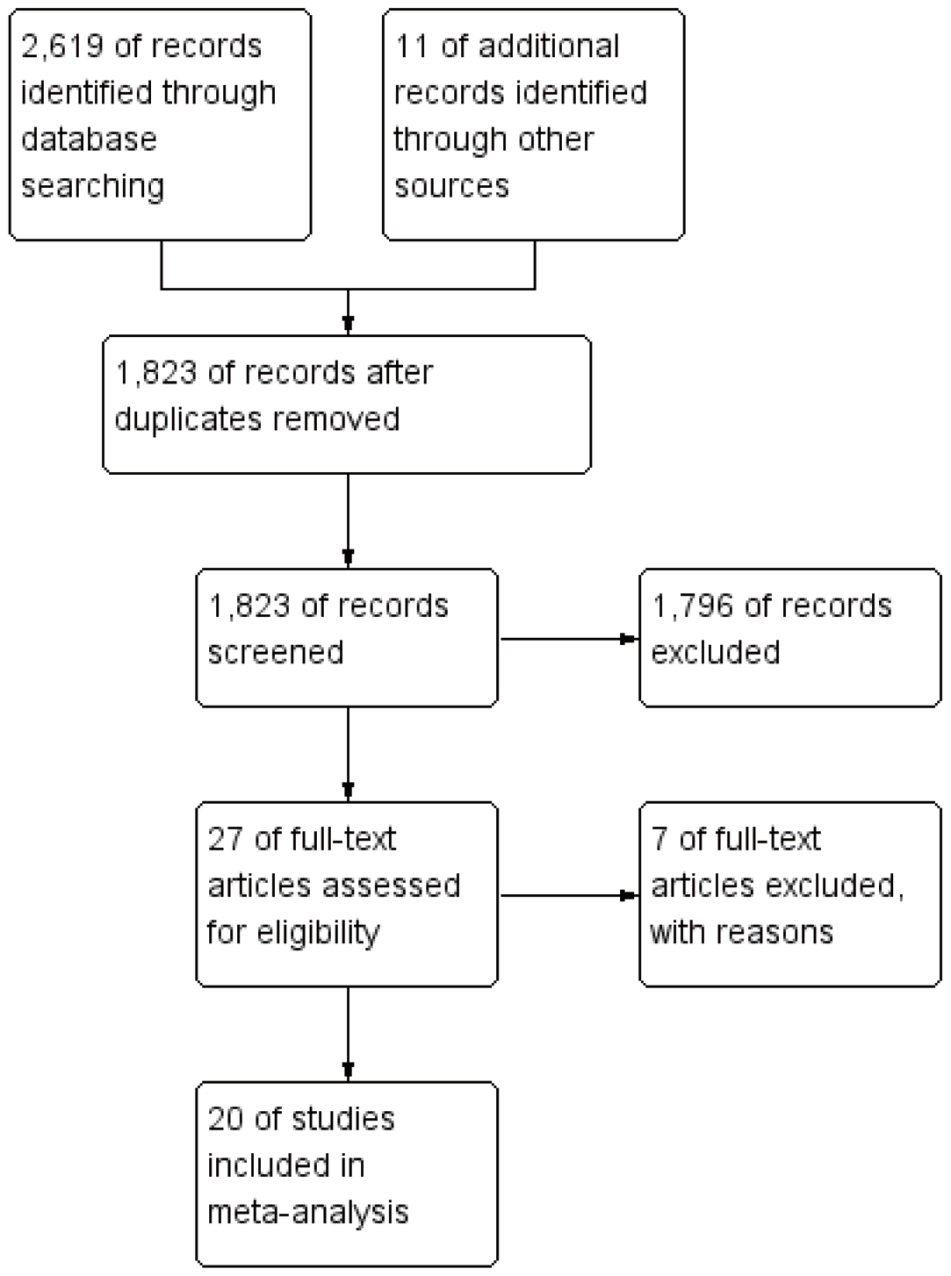
Flow diagram of screened, excluded, and analysed publications.

We identified 2,630 potentially relevant articles through database searching and other sources(shown in Fig 1). Of these, 2,603 articles were excluded after the first screening based on abstracts or titles, leaving 27 articles for full-text review. After further evaluation, five studies were excluded for lack of available data, and two studies were excluded because they were from the same population. At last, a total of 20 eligible studies published between 1996 and 2012 were identified, including ten case-control studies [20]–[29], four cohort studies [30]–[33], and six RCTs [34]–[39] (Baseline data and other details of included studies were shown in Table 1). A total of 1,139,584 subjects, including 15,297 haematological malignancies cases were involved. Of the 20 included studies, eight studies were conducted in Europe [20], [25], [27], [29], [33]–[36], nine studies in America [21]–[23], [26], [28], [31], [32], [38], [39], and remaining three studies in other countries [24], [30], [37]. Nine studies were hospital-based [21], [22], [24], [34]–[39], and 11 studies were population-based [20], [23], [25]–[33].

**Table 1:** Characteristics of included studies assessing the risk of haematological malignancies with statin use.

Study	Year of publication	Study design	Country	Statin	Follow up (years)	Time Period	Sex	Study setting	Cases/Subjects	Cancer outcome	Confounding variables adjusted
Lutski M	2012	cohort	Israel	A,P,S	4.7(mean)	1998–2006	M/F	Population-based	681/202,648	Haematological malignancies HR: 0.69 (0.55–0.88)Leukemia HR: 0.58 (0.37–0.91)Lymphoma HR: 0.69 (0.51–0.94)	Age, sex, marital status, area of residence, nationality, socioeconomic level, years of stay in Israel, obesity, diabetes mellitus, hypertension, cardiovascular disease, efficacy, hospitalizations and visits to physicians a year before first statin dispensation, and asthma
HPS	2011	RCT	England	S	5.3(mean)	1994–2001	M/F	Hospital-based	327/20,536	Haematological malignancies RR: 1.01 (0.81–1.25)	Randomization
Vinogradova Y	2011	case-control	England	A,P,S	2.3(median)	1998–2008	M/F	Population-based	7,185/29,162	Haematological malignancies OR: 0.78 (0.71–0.86)	Townsend quintile, BMI, smoking status, myocardial infarction, coronary heart disease, diabetes, hypertension, stroke, rheumatoid arthritis, use of NSAIDs, Cox2-inhibitors, aspirin
Jacobs EJ	2011	cohort	America	F,L,P,S	≥5(mean)	1997–2007	M/F	Population-based	1,005/133,255	Non-Hodgkin lymphoma RR: 0.74 (0.62–0.89)	Age, sex, race, education, smoking, use of NSAIDs, BMI, physical activity, history of elevated cholesterol, diabetes, heart disease, hypertension
Chao C	2011	case-control	America	A,L,P,S	NR	1996–2008	M/F	Hospital-based	259/1,554	Non-Hodgkin lymphoma HR: 0.55 (0.31–0.95)	Age, sex, race, index year, known duration of HIV infection, Kaiser Permanente region (Northern or Southern California), clinical AIDS diagnosis prior to index date (yes/no), duration of antiretroviral therapy (ART) use (years), baseline CD4 cell count level (<200, 201–500, and>500/m l), and history of selected co-morbidity (yes/no), history of hepatitis B and C, diabetes, and obesity
Friedman GD	2008	cohort	America	A, C, F, L, P, R, S	≥5(mean)	1994–2003	M/F	Population-based	312/361,859	Hodgkin lymphoma HR: 1.08 (0.26–4.42)Non-Hodgkin lymphoma HR: 1.02 (0.71–1.45)Multiple myeloma HR: 0.81 (0.42–1.58)Lymphocytic leukemia HR: 0.86 (0.41–1.84)Myeloid leukemia HR: 0.40 (0.15–1.09)	Smoking, use of NSAIDs, calendar year
Coogan PF	2007	case-control	America	NR	3–6(median)	1991–2005	M/F	Hospital-based	25/379	Leukemia OR: 1.1 (0.6–2.0)Non-Hogdkin lymphoma OR: 1.2 (0.6–2.4)	Age, sex, BMI, interview year, study center, alcohol consumption, race, years of education, smoking, use of NSAID
Landgren O	2006	case-control	America	NR	1.8–11.2(median)	1996–2002	F	Population-based	179/870	Multiple myeloma OR:0.4(0.2–0.8)	Age, race, education, and BMI
Iwata H	2006	case-control	Japan	F,P,S	4(median)	1995–2001	M/F	Hospital-based	221/1100	Lymphoma OR: 2.06(0.88–4.8)Multiple myeloma OR: 3.99(1.75–9.10)	Age, sex, year of visit, serological status for anti-Hepatitis B surface antigens (HBsAg) and anti-Hepatitis C virus antibodies (HCVAb)
Fortuny J	2006	case-control	Czech Republic, France, Germany, Ireland, Italy, and Spain		>6.25(mean)	1998–2004	M/F	Population-based	2,362/4,568	Lymphoma OR: 0.61 (0.33–1.15)	Age, gender, and country
Friis S	2005	cohort	Denmark	A, C, F, L, P, S	3.3(mean)	1989–2002	M/F	Population-based	1,626/334,754	Haematological malignancies RR: 0.88 (0.60–1.29)	Age, sex, calendar period, use of NSAIDs, use of hormone, use of cardiovascular drugs
Zhang Y	2004	case-control	America	NR	NR	1996–2000	F	Population-based	601/1,318	Non-Hodgkin lymphoma OR: 0.5(0.4–0.8)	Age, BMI, menopausal status, and family history of non-Hodgkin lymphoma
Strandberg TE	2004	RCT	Nordic countries	S	5.4(median)	1988–1994	M/F	Hospital-based	36/4,444	Haematological malignancies RR: 1.12 (0.58–2.14)	Randomization
Graaf MR	2004	case-control	Netherlands	A, C, F, P, S	7.2(mean)	1995–1998	M/F	Population-based	93/20,105	Lymphoma OR: 0.28 (0.06–1.30)	Age, sex, geographic region, follow-up time, calendar time, diabetes mellitus, chronic use of diuretics, use of ACE inhibitors,use of calcium antagonists, use of NSAIDs, use of hormones, other lipid-lowering therapies, familiar hypercholesterolemia
Holdaas H	2003	RCT	Belgium, Denmark, Finland, Germany, Norway,Sweden, Switzerland, the UK, and Canada	F	5.1(mean)	1996–1997	M/F	Hospital-based	29/2,102	Haematological malignancies RR: 0.61 (0.29–1.29)	Randomization
LIPID Study Group	2002	RCT	Australia and New Zealand	P	≥8(mean)	1990–1992	M/F	Hospital-based	89/7,680	Haematological malignancies RR: 0.70 (0.46–1.07)	Randomization
Blais L	2000	case-control	Canada	L, P, S	2.7(median)	1988–1994	M/F	Population-based	24/264	Lymphoma RR: 2.17 (0.38–12.36)	Age, sex, use of fibric acid, use of other lipid-reducing agents, previous benign neoplasm, year of cohort entry, the score of comorbidity
Downs JR	1998	RCT	America	L	5.2(mean)	1990–1997	M/F	Hospital-based	23/6,605	Lymphoma RR: 0.92 (0.41–2.08)	Randomization
Traversa G	1998	case-control	Italy	NR	NR	1992–1994	M/F	Population-based	202/2,222	Leukemia OR: 1.3 (0.6–3.0)	Age,gender
Sacks FM	1996	RCT	Canada and America	P	5(mean)	1989–1991	M/F	Hospital-based	18/4,159	Haematological malignancies RR: 0.80(0.32–2.02)	Randomization

NR = not reported; RR = Relative risk; HR = Hazard ratio; OR = Odds ratio; M =  male; F = female; BMI = body mass index; RCT =  randomized controlled trial.

### Risk of Haematological Malignancies

**Figure 2:**
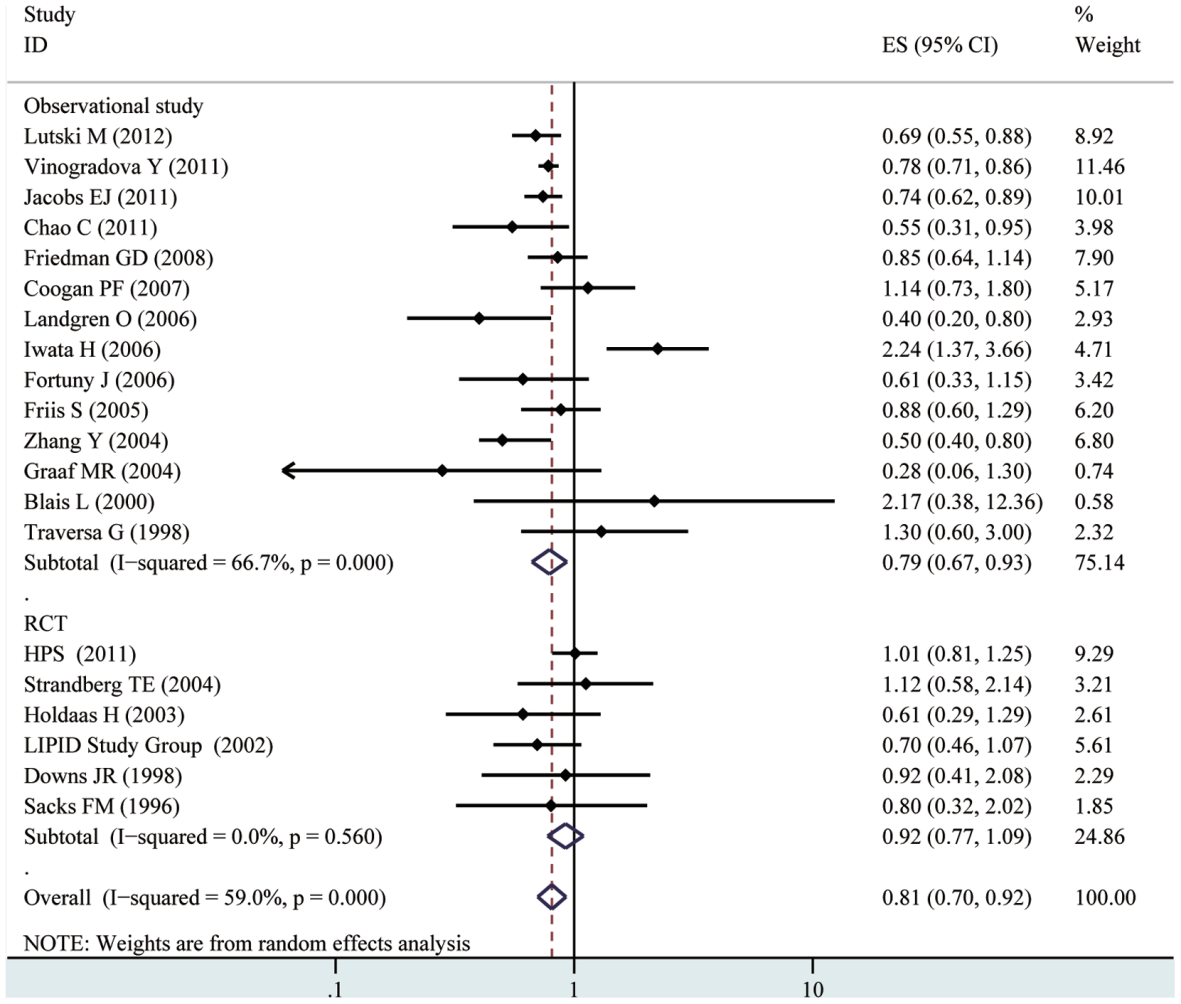
Forest plot: estimates (95% CIs) of statin use and risk of haematological malignancies. Squares indicated study-specific risk estimates (size of square reflects the study-statistical weight, i.e. inverse of variance); horizontal lines indicate 95% confidence intervals; diamond indicates summary relative risk estimate with its corresponding 95% confidence interval.

**Figure 3:**
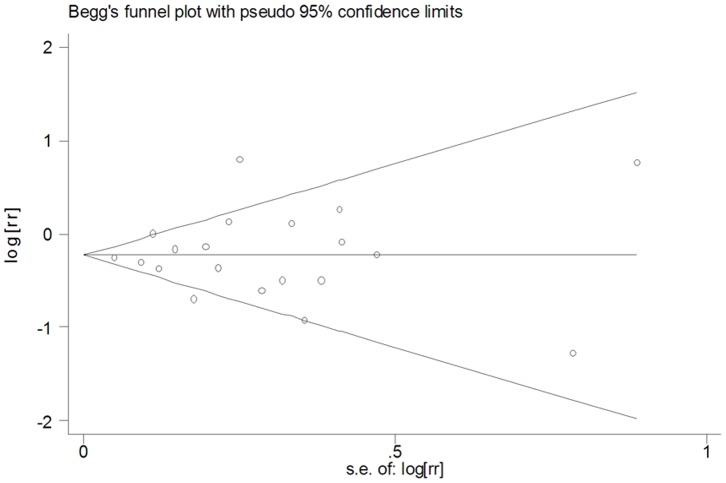
Funnel plot for publication bias in the studies investigating the association between statin use and the risk of haematological malignancies.

Based on data from 20 studies assessing the risk of haematological malignancies, the use of statins was associated with a statistically significant 19% reduction in haematological malignancies incidence (RR = 0.81, 95% CI [0.70, 0.92]). There was, however, considerable heterogeneity observed across studies (I^2^ = 59.0%, p<0.001). Both multivariable adjusted RR estimates with 95% CIs of each study and combined RR were shown in Fig.2. In the present meta-analysis, no publication bias was observed among studies using?Begg’s P value(P = 0.95); Egger’s(P = 0.78) test, which suggested there was no evidence of publication bias (Fig. 3).

### Subgroup Analysis

We carried out subgroup analyses of studies based on study design, study location, study setting, and subtypes of haematological malignancies (Table 2). Statin use was associated with a significantly reduced risk of haematological malignancies among observational studies(RR = 0.79, 95% CI [0.67, 0.93]), but not among RCTs(RR = 0.92, 95% CI [0.77, 1.09]). When stratified the various studies by study location, we found a significant association among studies conducted in Western countries (RR = 0.78, 95%CI [0.69, 0.88]), but not among studies conducted in Asian countries(RR = 1.22, 95%CI [0.38, 3.86]). When we examined whether the associations differed by study setting, statin use was significantly associated with a reduced risk of haematological malignancies among population-based studies(RR = 0.73, 95% CI [0.64, 0.83]), but not among hospital-based studies(RR = 0.96, 95% CI [0.73, 1.25]). When we stratified the various studies by cancer subtype, we found that statin therapy was associated with a significantly reduced risk of lymphoma(RR = 0.76, 95% CI [0.62, 0.95]), and a borderline significantly reduced risk of leukemia(RR = 0.77, 95% CI [0.57, 1.02]), but not multiple myeloma(RR = 0.86, 95% CI [0.19, 4.0]). We then divided the studies investigating statin use and risk of lymphoma to two subgroups(Non-Hodgkin lymphoma and Hodgkin lymphoma), and found that statin therapy was associated with a significantly reduced risk of Non-Hodgkin lymphoma(RR = 0.72, 95% CI [0.59, 0.87]), but not Hodgkin lymphoma(RR = 0.84, 95% CI [0.37, 1.95]).

**Table 2:** Subgroup analysis of all studies.

Grouping variable	Subgroups	No. of studies	Pooled estimate		Tests of heterogeneity	
			RR	95% CI	P value	I^2^(%)
All studies		20	0.81	0.70–0.92	<0.001	59.00
Study design	Observational study	14	0.79	0.67–0.93	<0.001	66.70
	RCT	6	0.92	0.77–1.09	0.56	0.00
Study location	Western countries	18	0.78	0.69–0.88	0.05	38.80
	Asian countries	2	1.22	0.38–3.86	<0.001	94.40
Study setting	Population-based	11	0.73	0.64–0.83	0.09	38.40
	Hospital-based	9	0.96	0.73–1.25	0.01	59.70
Cancer subtypes	Leukemia	4	0.77	0.57–1.02	0.19	37.50
	Lymphoma	11	0.76	0.62–0.95	0.02	51.60
	Non-Hodgkin lymphoma	6	0.72	0.59–0.87	0.04	55.80
	Hodgkin lymphoma	2	0.84	0.37–1.95	0.67	0.00
	Multiple myeloma	3	0.86	0.19–4.0	<0.001	90.10

No, number; RR, relative risks; CIs, confidence intervals; RCTs, randomized, controlled trials.

### Sensitivity Analysis and Meta-regression Analysis

To test the robustness of association and characterize possible sources of statistical heterogeneity, sensitivity analysis was carried out by excluding studies one-by-one and analyzing the homogeneity and effect size for all of the rest studies. Sensitivity analysis indicated that no significant variation in combined RR by excluding any of the study, confirming the stability of present results. To better investigate the possible sources of between-study heterogeneity, a meta-regression analysis was performed. Geographic area, publication year, follow-up time, study design, and study setting, which may be potential sources of heterogeneity, were tested by a meta-regression method. Finally, we found that study design and study setting had statistical significance in a multivariate model (P<0.05).

## Discussion

In this comprehensive meta-analysis of all existing studies(ten case-control studies, four cohort studies, and six RCTs) involving a total of 1,139,584 subjects with 15,297 cases of haematological malignancies, we found that statin use was inversely related to the risk for haematological malignancies, with a 19% reduction in the risk of haematological malignancies. There was statistically significant heterogeneity among the 20 included studies investigating the association between statin use and haematological malignancies risk, so a random-effect model was chosen over a fixed-effect model. Meta-regression analysis revealed that study design and study setting may be the source of heterogeneity. Our sensitivity analysis yielded similar and robust results, indicating that no study considerably influenced the overall risk estimate between statin use and haematological malignancies risk. Moreover, the results of Begg’ s test and Egger’ s test did not support the existence of major publication bias.

Previous in vitro studies have suggested anti-inflammatory and immunomodulatory properties of statins, including selective blockage of LFA-1-mediated adhesion and costimulation of lymphocytes [40], down regulation of class II major histocompatibility complexes on antigen-presenting cells [41], and reduction of chemokine synthesis in peripheral blood mononuclear cells [42]. In cell line and animal models, statins showed anticancer effects for haematological malignancies. Researchers have found that statins could induce apoptosis and inhibit proliferation of human acute myeloid leukemia cells and multiple myeloma cells [43]–[45]. Further, inhibitory effect of statins on spontaneous metastases derived from lymphoma was found in animal experiment [46]. So it is biologically plausible that statin use has protective effect upon haematological malignancies risk.

In our subgroup analyses, the results were substantially affected by study design. The chemopreventive effect of statins was seen primarily in observational studies, however, RCTs included in the present study did not demonstrate any significant chemopreventive effect of statins though there was a trend toward statistical significance (RR = 0.92, 95% CI [0.77, 1.09]). Importantly, the RCTs included in the meta-analysis were carried out mainly to investigate the effect of statins on cardiovascular morbidity. By design, the patients enrolled in these RCTs were not at high risk of development of haematological malignancies. And there were only six RCTs with a small number of participants and haematological malignancies cases, so it was not adequately powered to detect a significant difference in haematological malignancies incidence. Moreover, since the occurrence of cancer was not the primary objective of these trials, patients were not routinely screened for development of haematological malignancies; this might have affected the detection rate of haematological malignancies. These factors may explain why current clinical trials of statins did not demonstrate a statistically significant chemopreventive effect of statins against haematological malignancies. When stratified the various studies by study location, we found a significantly reduced risk in haematological malignancies among studies conducted in western countries, however, statin use had no significant association with haematological malignancies risk among studies conducted in Asian countries. The exact reason for the difference was unclear. The differences in genetic susceptibility, culture, and lifestyles may explain part of the inconsistency of the results. Further, we should notice that there were only two studies investigating the association between statin use and haematological malignancies risk. So more studies conducted in Asia are needed to confirm this association in the future. During subgroup analyses, we found that study setting also affected the association between statin use and haematological malignancies risk. A significant association was observed in population-based studies, but not in the hospital-based studies. The reason may be that the hospital-based studies have some inherent selection biases as such controls may just represent a sample of ill-defined reference population and may not be very representative of the study population or the general population. For the subgroup analysis of statin use and haematological malignancies risk by cancer subtype, we observed a statistically significant inverse association between statin use and Non-Hodgkin lymphoma, but not other subtypes of haematological malignancies, though there was a trend(we can see in Table2). More studies with more participants are needed to get a narrow confidence interval of RR and draw firm conclusions.

The strength of the present meta-analysis lies in a large sample size (1,139,584 subjects and 15,297 cases of haematological malignancies) and no significant evidence of publication bias. Two investigators independently performed the article identification, data extraction, and verification and resolved all discrepancies. Furthermore, our findings were stable and robust in sensitivity analysis. However, several limitations of this meta-analysis should be noted. Firstly, we did not search for unpublished studies, so only published studies were included in our meta-analysis. Therefore, publication bias may have occurred although no publication bias was indicated from both visualization of the funnel plot and Egger’s test. Secondly, the included studies were different in terms of study design and definition of drug exposure. Finally, the RCTs included in the meta-analysis were carried out mainly to investigate the effect of statins on cardiovascular morbidity. So, definitive, randomized chemoprevention trials are needed to more rigorously assess the effects of statins on incident haematological malignancies, but would be lengthy, logistically challenging and resource intensive.

Based on this comprehensive meta-analysis, statin use may have chemopreventive effects against haematological malignancies. More studies, especially definitive, randomized chemoprevention trials are needed to confirm this association.

## Supporting Information

Checklist S1 **PRISMA Checklist.**
(DOC)
